# Antibody and T Cell Immune Responses to SARS-CoV-2 Peptides in COVID-19 Convalescent Patients

**DOI:** 10.3389/fmicb.2022.842232

**Published:** 2022-04-18

**Authors:** Ekaterina Garanina, Shaimaa Hamza, Robert J. Stott-Marshall, Ekaterina Martynova, Maria Markelova, Yuriy Davidyuk, Venera Shakirova, Neha Kaushal, Manoj Baranwal, Ilsiyar M. Khaertynova, Albert Rizvanov, Toshana L. Foster, Svetlana Khaiboullina

**Affiliations:** ^1^Intitute of Fundamental Medicine and Biology, Kazan Federal University, Kazan, Russia; ^2^Faculty of Medicine and Health Sciences, School of Veterinary Medicine and Science, University of Nottingham, Loughborough, United Kingdom; ^3^Department of Infectious Diseases, Kazan State Medical University, Kazan, Russia; ^4^Department of Biotechnology, Thapar Institute of Engineering and Technology, Patiala, India

**Keywords:** SARS-CoV-2, COVID-19, spike protein, peptides, antibody humoral immune response

## Abstract

Identifying immunogenic targets of severe acute respiratory syndrome coronavirus-2 (SARS-CoV-2) is critical to advance diagnostic and disease control strategies. We analyzed humoral (ELISA) and T-cell (ELISpot) immune responses to spike (S) and nucleocapsid (N) SARS-CoV-2 proteins as well as to human endemic coronavirus (eCoV) peptides in serum from convalescent coronavirus disease 2019 (COVID-19) patients from Tatarstan, Russia. We identified multiple SARS-CoV-2 peptides that were reactive with serum antibodies and T cells from convalescent COVID-19. In addition, age and gender associated differences in the reactivity to S and N protein peptides were identified. Moreover, several SARS-CoV-2 peptides tested negatively correlated with disease severity and lung damage. Cross-reactivity to eCoV peptides was analyzed and found to be lower in COVID-19 compared to controls. In this study, we demonstrate the changing pattern of immunogenic peptide reactivity in COVID-19 serum based on age, gender and previous exposure to eCoVs. These data highlight how humoral immune responses and cytotoxic T cell responses to some of these peptides could contribute to SARS-CoV-2 pathogenesis.

## Introduction

In 2019, an outbreak of a “pneumonia of unknown etiology” in Wuhan province, China was linked to infection with severe acute respiratory syndrome coronavirus-2 (SARS-CoV-2) (Coronaviridae Study Group of the International Committee on Taxonomy of Viruses, 2020; WHO, 2020b). The World Health organization (WHO) subsequently announced the disease caused by SARS-CoV-2 as coronavirus disease 2019 (COVID-19) which has since been declared a pandemic (CDC, 2020; WHO, 2020a; Batra et al., 2021; CDC, 2021a). Although COVID-19 can be asymptomatic in a subset of patients, the disease is characterized by severe pneumonia, acute respiratory distress syndrome (Zhu et al., 2019; Grasselli et al., 2020; Oran and Topol, 2020) and could be fatal in older patients as well as in those with co-morbidities (Ruan et al., 2020; Wortham et al., 2020).

Studies on mild and severe SARS-CoV-2 infection have reported that the recovery from COVID-19 is dependent on the activation of antibody responses to SARS-CoV-2, where higher IgG antibody titers were found in patients with severe disease compared to those with mild or moderate forms of COVID-19 (Hu et al., 2020). Sun et al. (2020) have confirmed the role of humoral immune responses in the pathogenesis of SARS-CoV-2 by identifying spike (S) glycoprotein and nucleocapsid (N) proteins to be major immunogens. In a report by Röltgen et al. (2020) S-specific or receptor binding domain (RBD)-specific IgG antibody levels were observed to be higher in patients not admitted to the intensive care unit (ICU) whilst N-specific IgG levels were higher in ICU patients. A higher ratio of S-IgG/N-IgG associated with outpatients and a mild form of COVID-19 confirmed this finding (Röltgen et al., 2020). These data provide strong support for the role of antibody immune responses in pathogenesis of SARS-CoV-2.

Multiple studies have demonstrated early activation of the antibody responses to SARS-CoV-2 infection (Ma H. et al., 2020; Marklund et al., 2020; Shu et al., 2020), where both IgG and IgM antibodies are detected in patient sera. Interestingly, antibody titers progressively decline over the course of disease. Shu et al. (2020) reported that antibody titers peaked by day 18 followed by a subsequent decline, with IgM levels being the first to decrease below basal levels, however, IgG levels remained high. Similar data, published by Lou et al. (2020) and Zhang G. et al. (2020) demonstrated that a gradual decline of IgM occurred within the first month after the onset of disease, with IgG levels remaining elevated for several months. Indeed, multiple studies have shown that anti-SARS-CoV-2 IgM levels decline quickly, and although IgG levels remain elevated for several months, a gradual decline has been observed up to 7 months post-recovery suggesting that SARS-CoV-2 infection fails to elicit a long-term antibody response (Ibarrondo et al., 2020; Ripperger et al., 2020; Röltgen et al., 2020). The cause and duration of this decline in antibody titers remains unclear and calls for further studies on antibody dynamics in SARS-CoV-2 infected patients. Establishing the duration of antibody responses is particularly important in determining strategies to mitigate transmission, the likelihood of establishing “herd immunity” and in coordinating vaccination efforts.

S and N proteins have been identified as major SARS-CoV-2 antigens (Sun et al., 2020). Multiple epitopes have been identified on both proteins from studies using patient sera as well as from bioinformatic approaches (Grifoni et al., 2020a; Poh et al., 2020; Zheng and Song, 2020). In these studies, several predicted regions on the SARS-CoV-2 S protein containing T cell epitopes, having been found to be associated with a strong immune response (Gomez-Perosanz et al., 2020). Candidate targets for immune responses against SARS-CoV-2 have been predicted by Ahmed et al. (2020) and Grifoni et al. (2020b). Both studies have identified multiple S and N protein epitopes which could have a protective role against SARS-CoV-2 infection. As these epitope regions in the S and N proteins are conserved, it has been suggested that these identified epitopes could be a prospective target for vaccine development. In addition, epitope reactivity has been demonstrated with serum samples collected from COVID-19 patients in China, Switzerland, Singapore, and United States, with multiple, different epitopes identified as reacting with patient sera and plasma and thus used as markers for infection and disease severity (Amrun et al., 2020; Farrera-Soler et al., 2020; Shrock et al., 2020; Zhang B. Z. et al., 2020).

In this study we sought to demonstrate reactivity of COVID-19 serum from patients in Tatarstan, Russia, with SARS-CoV-2 S and N peptides previously identified as immunogenic. Also, we aimed to identify correlations of S and N peptide reactivity with clinical and demographic characteristics, severity of lung damage, age and duration of symptoms. Given the moderate sequence similarity between the structural and non-structural regions of different coronaviruses, we also sought to demonstrate COVID-19 convalescent sera cross-reactivity with human endemic coronavirus (eCoV) peptides. These findings could have implications for diagnostic assay development and assessment of vaccine efficacy.

## Materials and Methods

### Human Subjects

Convalescent serum samples were collected from 138 (38 ± 11.9 years old) COVID-19 patients (50 males and 102 females) and 39 age-matched controls (37.7 ± 14.3 years old, 18 males and 21 females) between March and December 2020. Furthermore, acute serum samples were collected from a separate group of 14 COVID-19 patients (55.6 ± 6.2 years old; 5 males and 9 females). Clinical characteristics were also collected for these patients (Table 1). The diagnosis of SARS-CoV-2 infection was established based on clinical presentation and was confirmed by qPCR. Convalescent serum samples were collected at various time points post-infection (0–12 months) following standard operating procedures in the hospital for the diagnosis of COVID-19 infection and stored at −80°C. Additionally, 22 (10 male and 12 female) control serum samples collected in 2015–2016 were included into the study. Aliquots (100 μL) of these serum samples were stored at −80°C. All controls were tested for anti-SARS-CoV-2 antibodies and were confirmed sero-negative.

**TABLE 1:** Clinical characteristics of acute and convalescent COVID-19 patients.

Clinical characteristics	Values
Age (years)	38 ± 0.05
Sex (M/F)	48/89
Lung damage (>40%) (n)	4
Lung damage (20–40%) (n)	23
Lung damage (<20%) (n)	110
Severe COVID-19 (n)	4
Moderate COVID-19 (n)	43
Mild COVID-19 (n)	105
Fever (°C)	37.92 ± 0.66
Fever (days)	6.31 ± 4.04
Artificial ventilation (yes/no)	2/135

*n = number of cases.*

In addition to serum samples, convalescent blood samples from 17 COVID-19 patients (35.9 ± 14.6 years old, 6 male and 11 female) as well as 9 controls (36.7 ± 14.5 years old; 5 male and 4 female) were collected.

### Ethics Statement

The Ethics Committee of the Kazan Federal University approved this study, and signed informed consent was obtained from each patient and control subjects according to the guidelines adopted under this protocol (Protocol 4/09 of the meeting of the Ethics Committee of the KSMA dated September 26, 2019). Sample collection in 2015–2016 was done according to the protocol approved by the Institutional Review Board of the Kazan Federal University and informed consent was obtained from each respective subject according to the guidelines approved under this protocol (Article 20, Federal Law “Protection of Health Right of Citizens of Russian Federation” N323-FZ, 11.21.2011).

### COVID-19 Peptides

Multiple S and N protein peptides for SARS-CoV-2 as well as eCoV NL63, OC43, HKU1 and 229E were synthesized by GenScript (Jiangsu, China). Amino acid (*aa*) sequences of SARS-CoV-2 S and N protein peptides were selected based on published data (Ahmed et al., 2020; Campbell et al., 2020; Grifoni et al., 2020a; Noorimotlagh et al., 2020). Also, S and N protein peptides for eCoVs were selected using the iedb.org platform (Vita et al., 2019).

**FIGURE 1:**
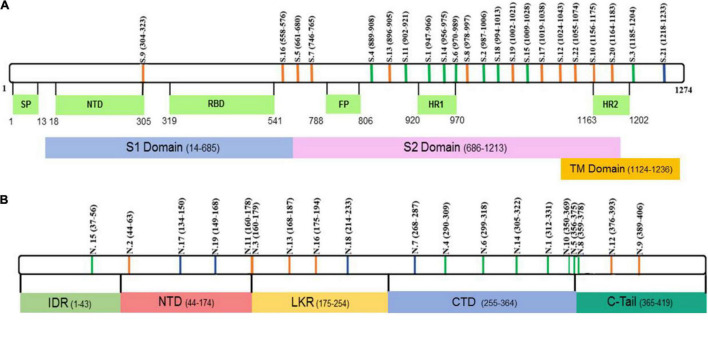
Schematic presentation of SARS-CoV-2 **(A)** S and **(B)** N peptide locations. Green – peptides containing both, B and T cell, epitopes. Blue – peptides containing only T cell epitopes. Orange – peptides containing only B cell epitopes.

SARS-CoV-2 S and N protein peptide aa sequences (purity > 95%) are summarized in Figure 1 and Supplementary Table 1. eCoV S and N proteins peptide aa sequences (purity > 95%) are summarized in Supplementary Table 2.

Alignment of S and N peptide aa sequences from SARS-CoV-2 with each eCoV [NL63, 229E, OC43, and HKU1 (GenBank Accession nos. YP_009724390, AKT07952, AWN62679, APU51916, YP_173238 for S protein and YP_009724397, YP_003771, NP_073556, YP_009555245, YP_173242 for N protein, respectively)] was done using the MegAlign program (Clustal W algorithm), DNASTAR software package Lasergene v. 7.1.0.44 (DNASTAR, Inc., United States) (Tamura et al., 2013). Parameters were adjusted manually.

### COVID-19 ELISA

The SARS-CoV-2-IgG IFA Best diagnostic ELISA kit (Vektor Best Agidel, Ufa, Russia) was used to determine SARS-CoV-2-specific antibody titers as per manufacturer’s instructions. Briefly, control or COVID-19 convalescent serum was diluted 1:100 with PBS and incubated for 60 min at 37°C in a 96-well plate with pre-adsorbed SARS-CoV-2 antigens. Following washes (3×; 0.5% Tween20 in PBS, PBS-T), wells were incubated with anti-human-IgG-HRP conjugated antibodies (1:10,000 in PBS-T, American Qualex Technologies, United States) for 30 min at 37°C. Post-incubation and washes (3×; PBS-T), wells were incubated with 3,3′,5,5′ Tetramethylbenzidine (Chema Medica, Moscow, Russia). The reaction was stopped by adding an equal amount of 10% phosphoric acid (TatKhimProduct, Kazan, Russia). Data were measured using a Tecan 200 microplate reader (Tecan, Switzerland) at OD_450_ with reference OD_650_. OD_450_ values higher than 0.5 were considered positive results, according to the manufacturer’s protocol.

### Peptide Reactivity With Serum Antibodies

Several peptides were selected for analysis of reactivity with SARS-CoV-2 infected patient as well as control subject sera. Each peptide (1 μg/100 μL) was adsorbed on a 384-well plate at 4°C for 18 h; plates were washed and incubated with serum samples (1:100; 50 μL) at 4°C for 18 h. Following washes (3×; PBS-T), wells were incubated with anti-human-IgG-HRP conjugated antibodies (1:10,000 in PBS-T, American Qualex Technologies, United States) for 30 min at 37°C. Washed (3×; PBS-T), wells were incubated with 3,3′,5,5′ Tetramethylbenzidine (Chema Medica, Moscow, Russia). The reaction was stopped by adding an equal amount of 10% phosphoric acid (TatKhimProduct, Kazan, Russia). Data were captured using a microplate reader Tecan 200 (Tecan, Switzerland) at OD_450_ with reference OD_650_.

### ELISpot Analysis

Peripheral blood mononuclear cells (PBMCs) were collected from COVID-19 convalescent individuals and used for ELISpot analysis. Leukocytes (10^5^ cells/well) were added to anti–human IFN-γ mAb (Abcam, United Kingdom) pre-coated 96-well plates in RPMI-1640 medium supplemented with 10% FBS (HyClone, South America), 2 mM L-glutamine (PanEco, Moscow, Russia) and 1% mixture of antibiotics penicillin-streptomycin (PanEco, Moscow, Russia). Peptides were added at 1 μg/well and plates incubated for 20 h (37°C, 5% CO_2_). Biotinylated mouse anti–human IFN-γ detection antibodies (clone 4S.B3; Abcam) were added for 90 min at room temperature followed by streptavidin alkaline phosphatase (1:1,000 dilution) for 60 min at room temperature. BCIP/NBT substrate solution [5-bromo-4-chloro-3-indolyl phosphate (BCIP)/nitro blue tetrazolium (NBT)] (Abcam, United Kingdom) was added for 25 min at room temperature. PBMCs incubated with phytohemagglutinin (PHA; 2.5 μg/ml) served as a positive control, while unstimulated leukocytes as a negative control. The number of spots in negative control wells (range of 0–5 spots) was subtracted from the number of spots in stimulated wells. Peptide stimulations were done in duplicate wells.

### Statistical Analysis

Statistical analysis was performed in the R environment (Dessau and Pipper, 2008). Statistically significant differences between comparison groups were accepted as *p* < 0.05, assessed by the Kruskal-Wallis test with Benjamini-Hochberg adjustment for multiple comparisons. Correlations were analyzed using the R psych package (based on Spearman’s rank correlation coefficient, *p*-values were adjusted with the Benjamini-Hochberg method).

## Results

### Clinical Presentation of COVID-19

A total of 152 samples (50 male and 102 female) were collected from COVID-19 acute and convalescent patients with an average age of 38.0 ± 11.9 years old (Table 1). Diagnosis of COVID-19 was established based on epidemiological anamnesis, clinical presentation and confirmed by qPCR. Clinical disease was diagnosed as mild (105 cases), moderate (43 cases) or severe (4 cases). Thirty-nine patients with moderate and 4 severe COVID-19 patients were hospitalized. All mild and four moderate COVID-19 patients did not require hospitalization. Mild COVID-19 was characterized as fever ≤ 38°C, cough, and throat pain. Patients with moderate COVID-19 had fever > 38°C, extent of lung damage as evaluated by computer tomography (CT) at stage 1–2 and oxygen saturation levels < 95%. In severe COVID-19 cases, fever was > 38°C, extent of lung damage by CT evaluated to be at stage 3–4, oxygen saturation levels < 93%, arterial pressure < 90/60 mmHg and some patients required artificial ventilation (Ministry of Health of the Russian Federation, 2020). Lung damage was categorized as < 20%, 20–40% or > 40% in 117, 31 and 4 patients, respectively. Fever was reported in all patients (37.92 ± 0.66°C) with duration of 6.31 ± 4.04 days. Two out of four of the severe COVID-19 patients required artificial ventilation, while none were admitted to the ICU. Control samples were collected from individuals who did not have symptoms of upper respiratory tract infection, did not have contact with COVID-19 patients and had negative anti-SARS-CoV-2 ELISA antibody test results. Control samples collected in 2015–2016 were also tested for anti-SARS-CoV-2 reactivity using ELISA tests and were found to be negative.

### Peptides Used in This Study

Peptide *aa* sequences were selected based on published data (Coronaviridae Study Group of the International Committee on Taxonomy of Viruses, 2020; Oran and Topol, 2020; WHO, 2020a,b). We selected peptides previously identified on SARS-CoV-2 N and S proteins which are essential for virus replication and binding to the ACE2 receptor (Shah et al., 2020; Supplementary Table 1). The position of these peptides is summarized in Figure 1. Interestingly, many peptides were found in regions identified as containing immunogenic epitopes (Liang et al., 2005; Shrock et al., 2020) suggesting that these peptides could have reactivity with COVID-19 patient cohort serum. There are multiple mutations that have been identified in circulating SARS-CoV-2 viruses (Callaway, 2020; Lauring and Hodcroft, 2021), raising serious concerns about vaccine efficacy and currently used diagnostic tools (Williams and Burgers, 2021). To address the possibility that some of the mutations are located in selected peptides, we have analyzed the location of mutations in currently circulating (release date: 1 July, 2021 to 16 August, 2021) nine delta strains and one reference strain (Supplementary Table 3) sequences for S and N protein of SARS-CoV-2. Mutation in S and N protein was assessed by taking reference sequences YP_009724390.1 and YP_009724397.2, respectively. Mutations in codons coding for sixteen and eight unique amino acids in S and N protein, respectively, were identified (Supplementary Table 4). Most of these mutations are outside of the S and N peptides selected for this study. Among mutations located in selected peptides, there were two S1 (D950N) and S22 (L1063F) located in S protein. Moreover, total of five [N2 (D63G), N18 (G215C), N10 and N5 (T362I), N8 (T362I, D377Y) and N12 (D377Y and R385K)], N protein peptides were found to contain mutations in currently circulating delta strains of SARS-CoV-2. These S and N peptides were found to have mostly a single mutation, which could have limited effect on their immunogenicity.

### Analysis of Antibody Response to COVID-19 Peptides Containing B-Cell Epitopes

**FIGURE 2:**
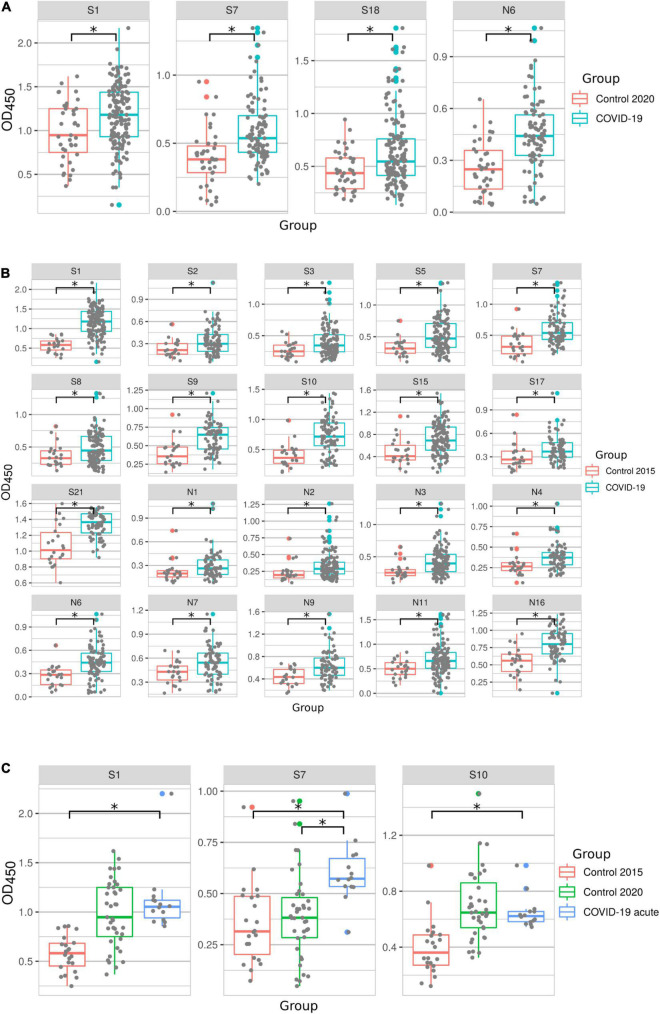
COVID-19 serum reactivity with SARS-CoV-2 peptides. Antibody reactivity was analyzed using ELISA. Peptides were adsorbed on a 384-well plate and probed with serum samples. Data is displayed for: **(A)** – peptides with increased reactivity in convalescent COVID-19 samples compared to controls collected in 2020; **(B)** – peptides with increased reactivity in convalescent COVID-19 samples compared to controls collected in 2015; **(C)** – peptides with increased reactivity in acute COVID-19 samples compared to controls collected in 2015 and 2020. Asterisks indicate statistically significant differences between reactivity to SARS-CoV-2 peptides (*p* < 0.05, Kruskal-Wallis test).

The reactivity of all 152 COVID-19 acute and convalescent serum samples was tested using B-cell peptides summarized in Figure 1 and Supplementary Table 1. Several S protein peptides (S1, S7, and S18) and the N6 peptide of N protein had significantly higher reactivity with convalescent serum compared to COVID-19 negative controls collected in year 2020 (Figure 2A). However, when compared with control samples collected in 2015, there were more SARS-CoV-2 peptides (S1, S2, S3, S5, S7, S8, S9, S10, S15, S17, S21, N1, N2, N3, N4, N6, N7, N9, N11, and N16) with higher reactivity with COVID-19 serum (Figure 2B). This observed difference between controls from 2015 and 2020 was due to lower reactivity of 2015 controls with SARS-CoV-2 peptides compared to 2020 controls. Similarly, more peptides were found to be reactive with acute COVID-19 serum when compared to 2015 in contrast to when compared to 2020 controls (S1, S7, and S10 peptides vs. S7 peptide) (Figure 2C). This could be explained by the priming of immune responses by endemic coronaviruses and thus the presence of cross-reacting antibodies in 2020 controls. This is further supported by the confirmation of all controls in this study having no previous exposure to SARS-CoV-2. Also, the effect of long-term storage on stability of serum content could contribute to variation in reactivity between 2015 and 2020 controls. Nevertheless, S1, S7, and N6 COVID-19 peptides had consistently high reactivity with patient serum as compared to controls from both 2015 and 2020, suggesting that these peptides could complement current diagnostic methods and could have therapeutic potential.

**FIGURE 3:**
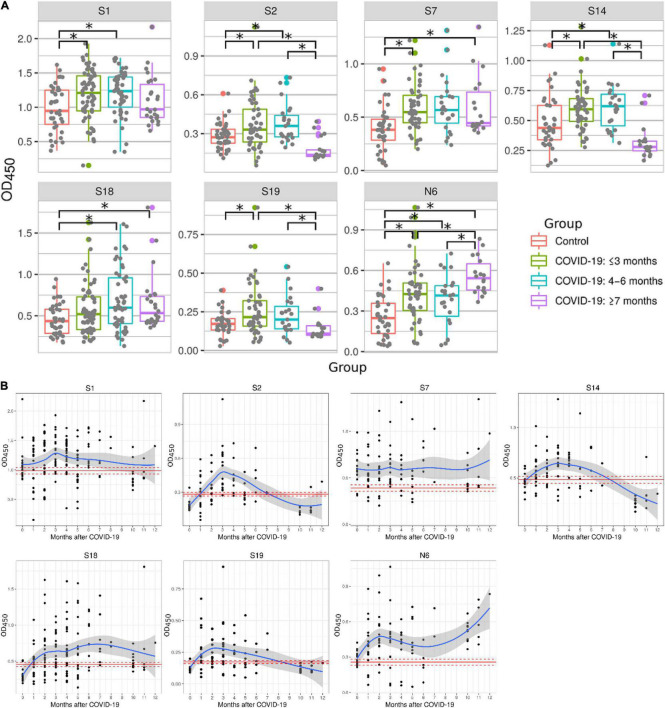
Reactivity of COVID-19 serum samples with SARS-CoV-2 peptides depending on time after convalescence. Antibody reactivity was analyzed using ELISA. Peptides were adsorbed on a 384-well plate and probed with serum samples. **(A)** Reactivity to SARS-CoV-2 peptides in COVID-19 serum samples grouped as: ≤3 months, 4–6 months and ≥7 months. Asterisks indicate statistically significant differences between reactivity to SARS-CoV-2 peptides depending on time since infection (*p* < 0.05, Kruskal-Wallis test with Benjamini-Hochberg adjustment). **(B)** Detailed dynamics of COVID-19 antibody reactivity with SARS-CoV-2 peptides. Red line- mean OD_450_ value in control; Dotted red line – mean ± standard error of mean of OD_450_ values in control; Blue line – local regression line (LOESS) of OD_450_ value in COVID-19; Gray area – standard error of mean for LOESS of OD_450_ values in COVID-19.

Studies have demonstrated that serum antibody levels declined in COVID-19 patients 6 months after recovery (Self et al., 2020; Seow et al., 2020). Therefore, we sought to determine reactivity of peptides with serum samples collected at different times after recovery (Figure 3A). Serum samples were separated into three groups: ≤3 months, 4–6 months and ≥ 7 months. Peptides S1, S2, S7, S14, S19, and N6 reacted significantly higher with serum samples collected ≤ 3 months after recovery when compared to 2020 control samples. Most of these peptides remained reactive 4–6 months post-recovery (S1, S2, S7, S14, S18, and N6). Reactivity with half of these peptides was lost later (≥ 7 months), when only S7, S18, and N6 peptides remained highly reactive in COVID-19 convalescent sera when compared to controls. These data demonstrate that reactivity to immunogenic epitopes is most versatile during early convalescence but declines with time after infection.

Next, we analyzed the dynamics of COVID-19 serum reactivity with SARS-CoV-2 peptides (Figure 3B). It appears that reactivity to some peptides (S1, S2 and S14, S19) was reduced few months after recovery. In contrast, reactivity to other peptides (S7, S18, and N6) remained high. Interestingly, reactivity to N6 was increasing with time post-recovery. These data suggest that antibody reactivity declines only to selected epitopes, not to all epitopes initially recognized.

### Sex-Based Differences in SARS-CoV-2 Peptide Reactivity

**FIGURE 4:**
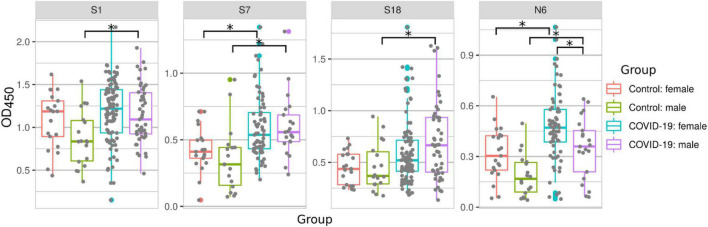
COVID-19 serum reactivity with SARS-CoV-2 peptides depending on sex of patient. Antibody reactivity was analyzed using ELISA. Peptides were adsorbed on a 384-well plate and probed with serum samples. Asterisks indicate statistically significant differences between reactivity to SARS-CoV-2 peptides depending on sex of the patient (*p* < 0.05, Kruskal-Wallis test with Benjamini-Hochberg adjustment).

It has been demonstrated that males may have more severe symptoms of COVID-19 and are at a higher risk of fatal outcome when compared to females (Kang and Jung, 2020; Tehrani et al., 2021). Therefore, we sought to determine whether serum reactivity to SARS-CoV-2 peptides differs depending on the sex of patients (Figure 4). We observed an increased serum reactivity with peptides S1, S7, S18, and N6 in male COVID-19 patients when compared with male controls. In female patients, serum reactivity was only higher for peptides S7 and N6 when compared to female controls (Figure 4). This highlights a difference in immune reactivity between male and female COVID-19 patients. A comparison of male and female COVID-19 samples showed an increased reactivity to N6 in female than in male COVID-19 patients.

### Age-Related Reactivity to SARS-CoV-2 S and N Protein Peptides

**FIGURE 5:**
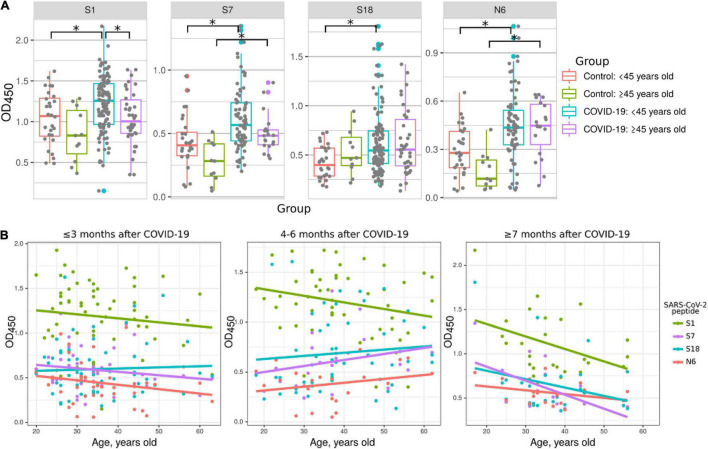
Reactivity of COVID-19 serum to SARS-CoV-2 peptides depending on age of patients. Patients were divided into young (<45 years old) and old (≥45 years old) groups. Antibody reactivity was analyzed using ELISA. Peptides were adsorbed on a 384-well plate and probed with serum samples. Only reactivity to peptides which significantly differed from that in corresponding age control is presented. **(A)** Age groups dependent difference to SARS-CoV-2 peptides reactivity in COVID-19 serum. Asterisks indicate statistically significant differences between reactivity to SARS-CoV-2 peptides depending on age of the patient (*p* < 0.05, Kruskal-Wallis test with Benjamini-Hochberg adjustment). **(B)** Age based distribution of reactivity to SARS-CoV-2 peptides in COVID-19 patient serum. Colored lines – linear regression of OD450 value.

Multiple studies have demonstrated that COVID-19 severity and fatality rates are increased in older patients (Wong et al., 2004; Li et al., 2020). We aimed to determine whether this age-related severity of COVID-19 could be explained by differences in antibody responses. COVID-19 convalescent samples were divided into younger (<45 years old) and older (≥45) groups (Figure 5) and used to analyze their reactivity to S1, S7, S18, and N6 peptides compared to corresponding controls. Interestingly, reactivity of younger patient serum to S1 was higher compared to that in older COVID-19 patients (Figure 5A). Also, the reactivity to peptides S7 and N6 was higher in both COVID-19 age groups compared to corresponding controls. However, only those in the <45 age group showed higher reactivity to peptides S1, and S18 compared to the same age control (Figure 5A). It appears that younger COVID-19 patients have higher reactivity with more peptides (S1, S7, S18, and N6) compared to older patients (S7 and N6). These data suggest that younger individuals having more COVID-19 reacting epitopes on S and N proteins is a contributing factor to age-related disease pathogenesis.

Next, we analyzed the age dynamics of antibody reactivity with S1, S7, S18, and N6 peptides, which demonstrated age-dependent difference (Figure 5B). This data clearly demonstrates that reactivity with peptides is higher in younger individuals. In contrast, with age, reactivity declines and, when over 50 years old, it becomes at the level of control. These data provide strong indication of more robust antibody response in younger as compared to that in older COVID-19 patients.

### Correlation Analysis of Serum Reactivity

**FIGURE 6:**
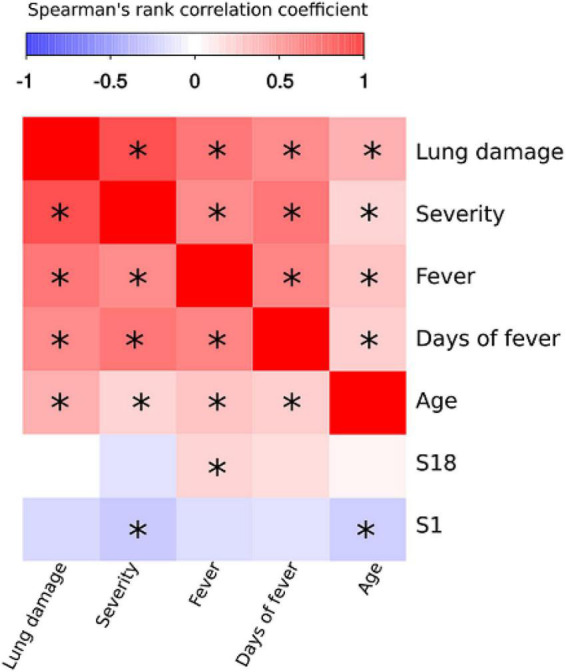
Correlation analysis of SARS-CoV-2 peptide antibody reactivity with clinical and demographic features of COVID-19. Spearman’s rank test was used to analyze correlation between reactivity with SARS-CoV-2 peptides and clinical presentation and characteristics of patients. Asterisks indicate statistically significant correlations (*p* < 0.05 with Benjamini-Hochberg adjustment).

We next sought to determine whether there is a correlation between serum reactivity with SARS-CoV-2 peptides as well as the clinical and demographic characteristics of the COVID-19 patients (Figure 6). Severity of COVID-19 positively correlated with increasing age, lung damage, high fever and duration of fever. There was also positive correlation between prolonged fever and lung damage (Figure 6). These data support previous findings and clinical observations in which older patients have more severe course of disease and higher mortality rate (CDC, 2021b; Sagar et al., 2021).

All peptides that demonstrated reactivity with COVID-19 convalescent serum were used for correlation analysis. Only S1 and S18 were identified as having statistically significant correlation with clinical parameters. Analysis of peptide reactivity and correlation with clinical presentation revealed significant negative correlation between severity and reactivity with S1 (Figure 6). We also observed that fever positively correlated with S18. Therefore, we suggest that negative correlation between reactivity with S1 peptide and severity of the disease could reflect its protective role in disease pathogenesis. Interestingly, S1 is located in HR1 region of S2 domain, which is also shown to be a potential target for novel anti-SARS-CoV-2 therapeutics (Liu et al., 2004).

### Analysis of T-Cell Response to SARS-CoV-2 T Cell Epitopes

Activation of T-cell immune responses during COVID-19 convalescence was analyzed using peptides containing T-cell epitopes (Figure 1 and Supplementary Table 1). Initially, peptides were screened for the most reactive and those that produced a minimum of two-fold increase compared with controls were selected for further study. Therefore, peptides having T cell epitopes and showing significant reactivity in preliminary screening (S4, S6, S15, N6, N10, and N19) were used in further analysis.

**FIGURE 7:**
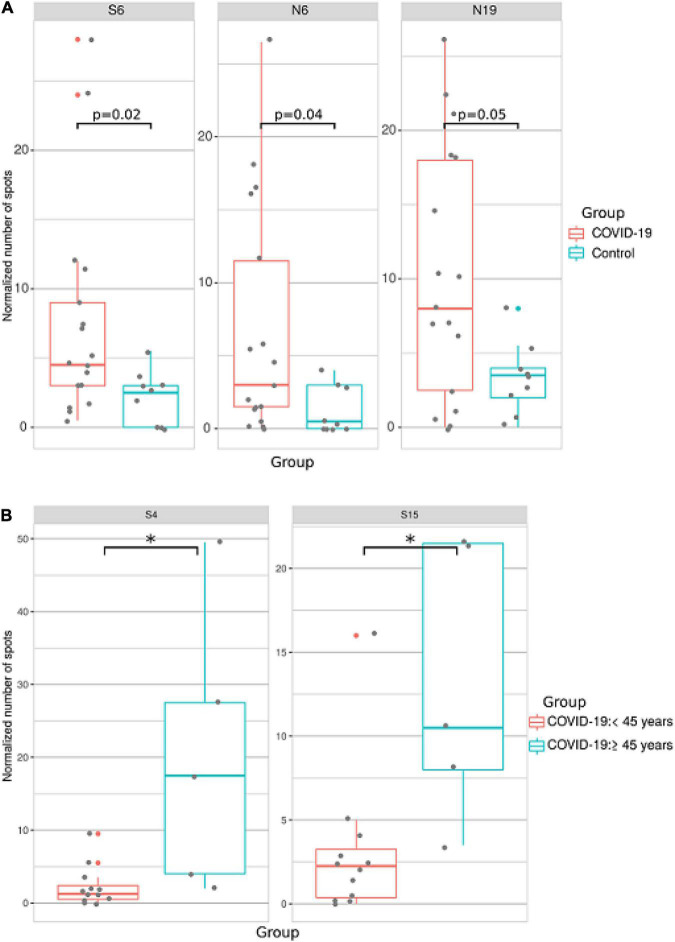
Analysis of T cell reactivity to SARS-CoV-2 peptides using ELISpot. PBMCs were collected from COVID-19 convalescent individuals and incubated with COVID-19 T cell peptides in anti–human IFN-γ mAb-coated 96-well plates. Spots were detected with biotinylated anti-human IFN-γ antibodies after 48 h. The number of spots in negative control wells was subtracted from the number of spots in stimulated wells. All experiments were done in duplicate. **(A)** Analysis of T cell reactivity to SARS-CoV-2 peptides in all COVID-19 patients. **(B)** Analysis of T cell reactivity to SARS-CoV-2 peptides in younger (<45 years old) and older (≤45 years old) COVID-19 patients. Asterisks indicate statistically significant differences between reactivity to SARS-CoV-2 peptides depending on age of the patient (*p* < 0.05, Kruskal-Wallis test).

Blood samples were collected from 17 COVID-19 patients and 9 controls. IFN-γ production by SARS-CoV-2 peptide-exposed T-cells was detected using the ELISpot method. We found that when activation of T-cells from all patients was analyzed independently of age, sex and time post-infection, reactivity to S6, N6, and N19 was significantly higher in COVID-19 patients when compared to controls (Figure 7A).

When ELISpot data was analyzed based on sex of the patient, we found no difference between male and female T-cell reactivity to SARS-CoV-2 T-cell peptides (Supplementary Table 5). When ELISpot data was analyzed by separating into two age groups (<45 and ≥45 years old), reactivity to S4 and S15 was higher in older compared to younger patients (Figure 7B).

### Analysis of Sequence Identity Between SARS-CoV-2 and Endemic Coronavirus Peptides

Studies have demonstrated cross reactivity between SARS-CoV-2 proteins and serum from non-SARS-CoV-2 infected patients (Kuxdorf-Alkirata et al., 2019; Ma Z. et al., 2020; Xie et al., 2020). This cross reactivity could be explained by the identity in *aa* sequence between SARS-CoV-2 peptides used in this study and the corresponding *aa* sequence in the same location in eCoV proteins (Supplementary Tables 6, 7). Silvanovitch et al. have demonstrated that immunological cross reactivity can occur when protein sequences are 70% conserved and higher (Graichen, 2021). Therefore, peptides with similarity greater than 70% between eCoVs and SARS-CoV-2 could contribute to immune cross reactivity. In this respect, ≥70% identity between SARS-CoV-2 S protein peptides (S1, S6, S8, S14, and S15) and the corresponding location on HKU1 and OC43 beta-coronaviruses (Table 2) suggests a contribution of these eCoVs to cross reactivity in COVID-19 sera. Interestingly, it appears that the S protein could potentially be the major contributor as none of the N protein peptides had ≥70% identity between SARS-CoV-2 and eCoVs. In contrast to beta-coronaviruses, only S protein peptides of NL63.S1 alpha-coronavirus had a 70% similarity (Supplementary Tables 8, 9), inferring a likely contribution of non-beta-coronaviruses to cross reactivity.

**TABLE 2:** Amino acid location displaying ≥70% identity between SARS-CoV-2 and eCoVs in S protein.

SARS-CoV-2 peptides	HKU1, S protein	OC43, S protein; APU51916		
		
	Location	Identity (%)	Location	Identity (%)
S1	1036–1055	73		
S6	1055–1074	72	1059–1078	80
S8	1063–1082	72	1067–1086	80
S14	1041–1060	73		
S15			859–865, 937–940, 958–961, 968–972	81
				

The location of eCoVs S and N immunogenic peptides used in this study differs from that of SARS-CoV-2 peptides. Therefore, we analyzed the identity of *aa* sequences of S and N peptides of eCoVs to the *aa* sequence of SARS-CoV-2 peptides in the same location (Supplementary Tables 8, 9). We found that the highest degree of identity was only between the aa sequence of NL63.S1 and the corresponding sequence of S protein of SARS-CoV-2. Other eCoV peptide identity with SARS-CoV-2 was less than 65%.

### Analysis of Antibody Reactivity With NL63, OC43, HKU1 and 229E Endemic Coronavirus Peptides

**FIGURE 8:**
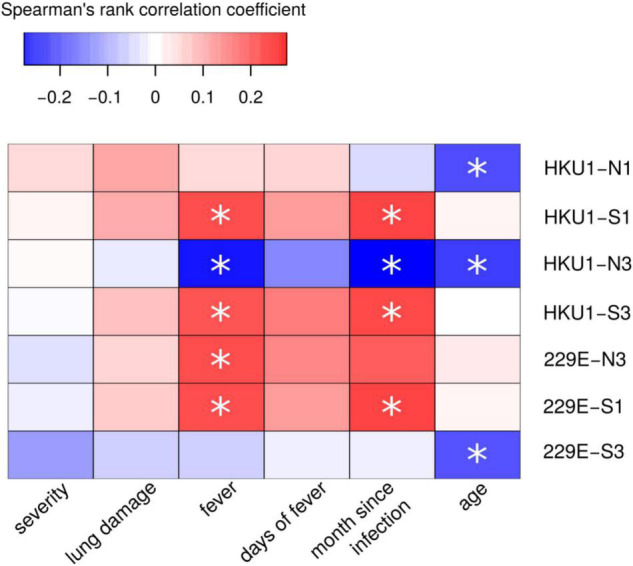
Correlation analysis of COVID-19 serum reactivity with eCoV peptides and demographic and clinical COVID-19. Spearman’s rank test was used to analyze correlation between reactivity with eCoV peptides and clinical presentation and characteristics of patients. Asterisks indicate statistically significant correlations (*p* < 0.05 with Benjamini-Hochberg adjustment).

Next, we sought to determine whether COVID-19 serum displayed cross-reactivity with eCoV peptides. ELISA analysis revealed mostly lack of difference in reactivity of COVID-19 patient serum with eCoV peptides when compared with 2020 control. The only difference was found in reactivity to HKU1-S1 peptide, though it was lower in COVID-19 serum when compared to the control (Supplementary Table 10). We next performed correlation analysis to determine if there was any relationship between eCoV peptide reactivity and severity of COVID-19 (Figure 8). While there was significant correlation between eCoV peptide reactivity and time since infection, there was no correlation with COVID-19 severity. This data suggests that prior exposure to eCoVs does not provide protection against severe forms of COVID-19.

## Discussion

The ongoing COVID-19 pandemic continues to present increasing healthcare, social and economic challenges worldwide, with the emergence of viral variants that may impact on vaccine efficacy and diagnostic development (Graichen, 2021; Seong et al., 2021). Analysis of immune responses facilitates identification of important viral targets, with potential for development of effective therapeutics. Use of convalescent plasma containing anti-SARS-CoV-2 IgG and IgM has been shown to be effective in treatment of severe and critical cases (Duan et al., 2020; Shen et al., 2020). Detection of anti-SARS-CoV-2 antibodies remains the main approach of diagnosis of previous exposure and efficacy of vaccination. Antibodies to S and N viral proteins have been demonstrated in convalescent serum; however, regions containing immunogenic epitopes in these proteins remain largely unknown. In the present study, we identified several peptides in SARS-CoV-2 S and N proteins with high reactivity to COVID-19 serum. Interestingly, S18 and N6 peptides were the most consistently reactive with convalescent serum up to 12 months after infection. These data suggest that N protein could also be used for detection of anti-SARS-CoV-2 antibodies, in addition to the more commonly used S protein.

Our data confirm the role of anti-SARS-CoV-2 S and N protein antibodies in the pathogenesis of COVID-19. Correlations between severity and antibody titer have been demonstrated in COVID-19 patients in multiple studies where both S and N proteins were shown to induce strong immune responses (Flemming, 2021; Hansen et al., 2021). The most common observation was a rapid decline of serum antibody titer in asymptomatic and mild COVID-19 cases (Flemming, 2021). Also, high levels of IgM and IgG were commonly found in severe compared to mild COVID-19 patients (Wang et al., 2020; Zhang et al., 2020). Despite this information, the immunogenic epitopes with protective capacity still remain largely unknown. Here, we identified multiple peptides on S and N proteins reacting with COVID-19 serum and, importantly, reactivity with these peptides negatively correlated with disease severity and lung damage. We found significant, albeit weak, negative correlation between COVID-19 severity and antibody reactivity with peptide S1. These data indicate that antibodies to this S protein region could have a protective role in SARS-CoV-2 infection.

It has been demonstrated that the severity of COVID-19 is higher in males than in females (Jin et al., 2020; Peckham et al., 2020). Several hypotheses have been proposed to explain sex-dependent differences in COVID-19 disease outcomes, where rapid and early development of antibody response has been linked to female sex hormones and X chromosomal factors (Furman et al., 2014; Flanagan et al., 2017). Takahashi et al. (2020) also demonstrated sex differences in immune responses to SARS-CoV-2 infection, with females having a higher proportion of circulating, activated and differentiated T-cells when compared to males. We further advanced our understanding of immune mechanisms in male and female COVID-19 patients by demonstrating differences in the reactivity with S and N SARS-CoV-2 peptides depending on the sex of the patient. Interestingly, reactivity to only S7 and N6 was found in female patients, whilst four peptides (S1, S7, S18, and N6) were identified as reacting with male COVID-19 serum. Although two peptides were common between both sexes (S7 and N6), the degree of reactivity for N6 was higher for female COVID-19 serum. Interestingly, convalescent serum reactivity with N protein was demonstrated in several studies (Brochot et al., 2020; Kang et al., 2021; Shah et al., 2021). The region between 200 and 419 aa appears to contain the commonly reactive epitopes (Pajenda et al., 2021). Supporting this observation is our finding of reactivity with N6 peptide, located at 299–318 aa. N protein has multiple functions such as binding and packaging virus RNA (Yao et al., 2020; Zeng et al., 2020) and has been shown to suppress host defense by interfering with type 1 IFN (IFN-1) signaling (Zhao et al., 2021). Therefore, anti-N protein antibodies could have a protective role by interfering with virus replication and preventing inhibition of the IFN-1 pathway. Additionally, data by Kang et al. (2021), where anti-N protein antibodies prevent complement activation, presented another protective mechanism for anti-N protein antibodies. This data suggests that sex differences in COVID-19 outcomes could depend on the quality of the immune response, rather than reactivity to a particular peptide.

We observed that younger patents had more antibody reactivity to S and N peptides. This suggests more epitopes are recognized in younger patients, which could lead to a more robust immune response to infection. Age-related differences in reactivity to SARS-CoV-2 peptides were also demonstrated in control subjects. Serum from young control subjects had higher reactivity with several SARS-CoV-2 peptides compared to older control subjects. Several studies have shown that mild or asymptomatic COVID-19 is more often diagnosed in younger compared to older individuals (Kang and Jung, 2020; O’Driscoll et al., 2021).

We have identified SARS-CoV-2 peptides containing T cell epitopes. Also, some peptides had higher reactivity in older (≥45 years old) compared to young (<45 years old) patients. As older patients are at higher risk of developing a severe form of COVID-19, the role of T-cells in age related clinical manifestation of disease could be suggested here. Multiple T-cell specific peptides were derived from S and N proteins and identified as immune activity in several studies (Ahmed et al., 2020; Grifoni et al., 2020a; Noorimotlagh et al., 2020). Interestingly, the location of the S6 (970–989aa) SARS-CoV-2 peptide, identified by Noorimotlagh et al. (2020) and containing T-cell epitope 976–984aa, is adjacent to another T-cell epitope 1000–1008aa, identified by Shomuradova et al. (2020). Here, we showed that COVID-19 serum had increased reactivity with peptide S6. Therefore, we suggest that S protein peptide 976–1008aa contains several SARS-CoV-2 T cell epitopes with high specificity.

A study by Meyerholz and Perlman has suggested a link between previous exposure to eCoVs and reduced severity of COVID-19 (Sagar et al., 2021). In contrast, Anderson et al. demonstrated that presence of the seasonal human coronavirus antibodies is not associated with protection against COVID-19 (Anderson et al., 2021). In another study it was suggested that SARS-CoV-2 infection can boost production of previously existing anti-eCov antibodies which are cross reactive, however, poorly specific and not neutralizing (Aguilar-Bretones et al., 2021). Our analysis revealed that SARS-CoV-2 infection has limited effect on antibody reactivity to eCoV peptides. Additionally, we found no correlation between antibody reactivity to eCoV and severity of COVID-19. Therefore, our data supports the assumption of the limited effect of existing anti-eCoV antibodies on clinical presentation of COVID-19. There is a potential limitation of our study as we were using selected eCoV peptides. Additionally, some sampling bias may affect these results as we analyzed samples from symptomatic COVID-19 patients. If previous exposure to eCoVs provided protection against SARS-CoV-2 infection, it is possible that samples from asymptomatic cases of COVID-19 would provide this evidence when compared with symptomatic cases. Also, there is a potential role of cross-reacting eCoV T cells in pathogenesis of COVID-19 which was suggested in several studies (da Silva Antunes et al., 2021; Quiros-Fernandez et al., 2021). Findings of the higher level of eCoV reactive T cells in individuals not exposed to SARS-CoV-2 and unvaccinated individuals (da Silva Antunes et al., 2021; Quiros-Fernandez et al., 2021), provide evidence suggesting the role of these cells in pathogenesis of COVID-19. Therefore, role of the immune response to eCoV in pathogenesis of COVID-19 could be complicated and involve antibody and T cell reactivity.

In conclusion, our data confirm early activation and circulation of anti-SARS-CoV-2 antibodies; though, it steadily declined with time post-infection. We also identified higher reactivity with several S and N peptides in younger patients compared with those over 45 years old, suggesting a contribution of reactivity with these epitopes to age-related pathogenesis. Several SARS-CoV-2 peptides negatively correlated with disease severity and lung damage. Moreover, our data demonstrate that COVID-19 serum has limited cross reactivity with eCoV peptides; however, there was no relationship between reactivity to eCoV peptides and severity of the disease. Taken together these findings identify several peptides from SARS-CoV-2 S and N proteins that are immunogenic and may be indicative of disease outcomes. Our data therefore underline the importance of both SARS-CoV-2 S and N regions in identifying T-cell epitopes and their potential for the development of prophylactic and therapeutic measures.

## Data Availability Statement

The original contributions presented in the study are included in the article/Supplementary Material, further inquiries can be directed to the corresponding authors.

## Ethics Statement

The studies involving human participants were reviewed and approved by the Ethics Committee of the Kazan Federal University approved this study, and signed informed consent was obtained from each patient and control subjects according to the guidelines adopted under this protocol (Protocol 4/09 of the meeting of the Ethics Committee of the KSMA dated September 26, 2019). Sample collection in 2015–2016 was done according to the protocol approved by the Institutional Review Board of the Kazan Federal University and informed consent was obtained from each respective subject according to the guidelines approved under this protocol (Article 20, Federal Law “Protection of Health Right of Citizens of Russian Federation” N323-FZ, 11.21.2011). The patients/participants provided their written informed consent to participate in this study.

## Author Contributions

EG and SK: conceptualization. SH, RS-M, and EM: investigation. SH and RS-M: formal analysis. MM and NK: visualization. NK and AR: funding acquisition. EM, MM, YD, VS, and NK: supervision. MB, IK, AR, TF, and NK: writing – original draft. YD, VS, EG, SH, RS-M, TF, SK, and AR: writing, review, and editing. All authors have read and agreed to the published version of the manuscript.

## Conflict of Interest

The authors declare that the research was conducted in the absence of any commercial or financial relationships that could be construed as a potential conflict of interest.

## Publisher’s Note

All claims expressed in this article are solely those of the authors and do not necessarily represent those of their affiliated organizations, or those of the publisher, the editors and the reviewers. Any product that may be evaluated in this article, or claim that may be made by its manufacturer, is not guaranteed or endorsed by the publisher.
