# Supplementary material for: Antibody and T Cell Immune Responses to SARS-CoV-2 Peptides in COVID-19 Convalescent Patients
Source: Front Microbiol. 2022 Apr 18;13:842232. doi: 10.3389/fmicb.2022.842232 (PMC9058163; doi:10.3389/fmicb.2022.842232)
Supplement: Supplementary file 1 [file Data_Sheet_1.docx]

Supplemental Table 1: SARS-CoV-2 spike (S) and nucleocapsid (N) peptide aa sequences

| peptide | aa sequence | Position (aa) | B epitope | T epitope | Resource |
| --- | --- | --- | --- | --- | --- |
| S1 | KLQDVVNQNAQALNTLVKQL | 947-966 | DVVNQNAQALNTLVKQL | ALNTLVKQL | [25] |
| S2 | VEAEVQIDRLITGRLQSLQT | 987-1006 | LITGRLQSL | EAEVQIDRLITGRLQSL | [25] |
| S3 | RLNEVAKNLNESLIDLQELG | 1185-1204 | RLNEVAKNLNESLIDLQELG | NLNESLIDL; RLNEVAKNL | [25] |
| S4 | GAGAALQIPFAMQMAYRFNG | 889-908 | GAALQIPFAMQMAYRF | GAALQIPFAMQMAYRFN | [25] |
| S5 | ECDIPIGAGICASYQTQTNS | 661-680 | GAGICASY |  | [30] |
| S6 | FGAISSVLNDILSRLDKVEA | 970-989 | AISSVLNDILSRLDKVE | VLNDILSRL | [30] |
| S7 | STECSNLLLQYGSFCTQLNR | 746-765 | GSFCTQLN |  | [30] |
| S8 | NDILSRLDKVEAEVQIDRLI | 978-997 | ILSRLDKVEAEVQIDRL |  | [30] |
| S9 | KSFTVEKGIYQTSNFRVQPT | 304-323 | KGIYQTSN |  | [30] |
| S10 | FKNHTSPDVDLGDISGINAS | 1156-1175 | KNHTSPDVDLGDISGIN |  | [30] |
| S11 | MAYRFNGIGVTQNVLYENQK | 902-921 | MAYRFNGIGVTQNVLYE | MAYRFNGIGVTQNVLY | [24] |
| S12 | LAATKMSECVLGQSKRVDFC | 1024-1043 | AATKMSECVLGQSKRVD |  | [30] |
| S13 | IPFAMQMAYRFNGIGVTQNV | 896-915 | PFAMQMAYRFNGIGVTQ |  | [30] |
| S14 | AQALNTLVKQLSSNFGAISS | 956-975 | QALNTLVKQLSSNFGAI | QALNTLVKQLSSNFGAI | [24, 25] |
| S15 | TQQLIRAAEIRASANLAATK | 1009-1028 | QLIRAAEIRASANLAATK | QLIRAAEIRASANLAAT | [25] |
| S16 | KFLPFQQFGRDIADTTDAV | 558-576 | QQFGRD |  | [30] |
| S17 | RASANLAATKMSECVLGQSK | 1019-1038 | RASANLAATKMSECVLG |  | [74] |
| S18 | DRLITGRLQSLQTYVTQQLI | 994-1013 | RLITGRLQSLQTYVTQQ | LITGRLQSL | [25] |
| S19 | QSLQTYVTQQLIRAAEIRAS | 1002-1021 | SLQTYVTQQLIRAAEIR |  | [30] |
| S20 | VDLGDISGINASVVNIQKEI | 1164-1183 | DLGDISGINASVVNIQK |  | [25] |
| S21 | LGFIAGLIAIVMVTIM | 1218-1233 |  | FIAGLIAIV | [25, 31] |
| S22 | SAPHGVVFLHVTYVPAQEKN | 1055-1074 | VVFLHVTYV |  | [25] |
| N1 | SAFFGMSRIGMEVTPSGTWL | 312-331 | FFGMSRIGMEVTPSGTW | AFFGMSRIGMEVTPSGTW; MEVTPSGTWL; GMSRIGMEV | [25] |
| N2 | GLPNNTASWFTALTQHGKED | 44-63 | GLPNNTASWFTALTQHGK |  | [74] |
| N3 | QLPQGTTLPKGFYAEGSRGG | 160-179 | GTTLPK; QLPQGTTLPKGFYAE; QLPQGTTLPKGFYAEGSR |  | [74] |
| N4 | ELIRQGTDYKHWPQIAQFAP | 290-309 | IRQGTDYKHWPQIAQFA; QGTDYKHW | IRQGTDYKHWPQIAQFA | [74] |
| N5 | HIDAYKTFPPTEPKKDKKKK | 356-375 | KHIDAYKTFPPTEPKKDKKK; TFPPTEPK | YKTFPPTEPKKDKKKK | [74] |
| N6 | KHWPQIAQFAPSASAFFGMS | 299-318 | KHWPQIAQFAPSASAFF | KHWPQIAQFAPSASAFF | [74] |
| N7 | YNVTQAFGRRGPEQTQGNFG | 268-287 |  | YNVTQAFGRRGPEQTQGNF | [74] |
| N8 | AYKTFPPTEPKKDKKKKADE | 359-378 | KTFPPTEPKKDKKKK | YKTFPPTEPKKDKKKK | [74] |
| N9 | QQTVTLLPAADLDDFSKQ | 389-406 | LLPAAD |  | [74] |
| N10 | VILLNKHIDAYKTFPPTEPK | 350-369 | LNKHIDAYKTFPPTEPK | ILLNKHID; ILLNKHIDA; LLNKHIDAYKTFPPTEPK | [74] |
| N11 | QLPQGTTLPKGFYAEGSRG | 160-178 | LPQGTTLPKG; QLPQGTTLPKGFYAE; QLPQGTTLPKGFYAEGSR |  | [74] |
| N12 | ADETQALPQRQKKQQTVT | 376-393 | LPQRQKKQ |  | [74] |
| N13 | PKGFYAEGSRGGSQASSRSS | 168-187 | PKGFYAEGSRGGSQASSRSS |  | [74] |
| N14 | AQFAPSASAFFGMSRIGM | 305-322 | QFAPSASAFFGMSRIGM | AQFAPSASAFFGMSR; AQFAPSASAFFGMSRIGM | [74] |
| N15 | SKQRRPQGLPNNTASWFTAL | 37-56 | RRPQGLPNNTASWFT | RRPQGLPNNTASWFT | [74] |
| N16 | GSRGGSQASSRSSSRSRNSS | 175-194 | SQASSRSS; SRGGSQASSRSSSRSR |  | [74] |
| N17 | ATEGALNTPKDHIGTRN | 134-150 |  | ALNTPKDHI | [74] |
| N18 | GGDAALALLLLDRLNQLESK | 214-233 |  | LALLLLDRL; LLLDRLNQL | [74] |
| N19 | RNPANNAAIVLQLPQGTTLP | 149-168 |  | LQLPQGTTL | [74] |

Supplemental Table 2: Human endemic coronavirus (eCoV) (HKU1, OC43, NL63, 229E) peptide aa sequences

| peptide | aa sequence | B epitope | T epitope | Genbank ID |
| --- | --- | --- | --- | --- |
| HKU1.N1 | RSGILKKTSWADQSERNYQT | SWADQSERNYQT | RSGILKKTSWADQSERNY | YP_173242.1 |
| HKU1.N2 | AFGVPPSEAKGYWYRHSRRS | AFGVPPSEAKGY | YWYRHSRRSF |  |
| HKU1.N3 | SNDFTPEDHSLLATLDDPYV | SNDFTPED | LLATLDDPYV |  |
| HKU-1.S1 | YNSPSSSSSRRKRRSISASY | YNSPSSSSSRRKR | ISASYRFVTF | YP_173238.1 |
| HKU-1.S2 | DHTDWCRCSCLPDPITAYDP | DPITAYDPRSCSQ | DHTDW |  |
| HKU-1.S3 | ATVAAMFPPWSAAAGIPF | PPWSAAAG | PWSAAAGIPF |  |
| OC43.N1 | FVEGQGVPIAPGVPATEAKG | GQGVPIAPGVPATEAKG | FVEGQGV | YP_009555245.1 |
| OC43.N2 | ADQSDQFRNVQTRGRRAQPK | DQSDQFRN | ADQSDQFRNVQTRGRRAQPK |  |
| OC43.N3 | RLELAKVQNLSGNPDEPQKD | NLSGNPDEPQKD | RLELAKVQNLSG |  |
| OC43.S1 | DVYRRKPDLPNCNIEAW | DVYRRKPDLPNC | LPNCNIEAW | APU51916.1 |
| OC43.S2 | GEHCSGLAVKSDYCGNNSCT | DYCGNNSCTC | GEHCSGLAV |  |
| OC43.S3 | CTLDPITLKAPDTYKCPQSK | LKAPDTYKCPQSK | CTLDPI |  |
| NL63.N1 | RVIPRNLVPIGKGNKDEQI | PIGKGNKDEQI | RVIPRNLVP | YP_003771.1 |
| NL63.N2 | EGAKTVNTSLGNRKRNQKPL | EGAKTVNTSLGNRKRNQKPL | EGAKTVNTSL |  |
| NL63.N3 | EVSTDEVGDNVQITYTYKM | EVSTDEVGDNV | VQITYTYK |  |
| NL63.S1 | NWADDRAARKKFPPPSFYMP | NWADDRAARKKFPPPSFYMP | RAARKKFP | AKT07952.1 |
| NL63.S2 | ISNSIQAIYDRLDSIQADQQ | SIQADQQ | ISNSIQAIY |  |
| NL63.S3 | GYTDNIFSVQQDGRIPNGFP | PNGFP | YTDNIFSVQQ |  |
| 229E.N1 | TKEQKHEMQKPRWKRQPNDD | TKEQKHEMQKPRWKRQPNDD | EMQKPRWKR | NP_073556.1 |
| 229E.N2 | WADASEPQRGRQGRIPYSLY | WADASEPQRGR | RQGRIPYSLY |  |
| 229E.N7 | VVEEPDSRAPSRSQSRSQSR | VVEEPDSRAPSRSQSRSQSR | RSQSRSQSR |  |
| 229E.S1 | LGTVDADYKKCTKGLSIADL | LGTVDADYKK | CTKGLSIADL | AWH62679.1 |
| 229E.S2 | SLPRSGSRVAGRSAIEDILF | SLPRSGSRVAGRS | YNLSSVIPSL |  |
| 229E.S3 | YNLSSVIPSLPRSGSRVAGR |  | EQYNQTILNLTSEISTLENK |  |

S and N protein peptides for eCoVs were selected using the iedb.org platform [32]. Genbank ID – is the source of eCoVs used for selection of immunogenic S and N protein peptides.

Supplemental table 3: Unique sequences of delta and delta plus strain for S and N protein

| **Accession No.** | **Strain** | **Release date** |
| --- | --- | --- |
| **S protein** | | |
| QYF06172 | B.1.617.2 (Delta strain) | 02-08-2021 |
| QXP40573 | B.1.617.2 (Delta strain) | 19-07-2021 |
| QXN17599 | B.1.617.2 (Delta strain) | 14-07-2021 |
| QYY14209 | B.1.617.2 (Delta strain) | 16-08-2021 |
| QYY15257 | B.1.617.2 (Delta strain) | 16-08-2021 |
| QYZ77148 | B.1.617.2 (Delta strain) | 16-08-2021 |
| QYJ09458 | AY.1 (Delta plus strain) | 04-08-2021 |
| QYY19368 | AY.2 (Delta plus strain) | 16-08-2021 |
| QYY74568 | AY.2 (Delta plus strain) | 16-08-2021 |
| **N protein** | | |
| QYY13729 | B.1.617.2 (Delta strain) | 16-08-2021 |
| QXL79997 | B.1.617.2 (Delta strain) | 14-08-2021 |
| BDA52120 | B.1.617.2 (Delta strain) | 12-08-2021 |
| QYU36587 | B.1.617.2 (Delta strain) | 12-08-2021 |
| QYK35657 | B.1.617.2 (Delta strain) | 05-08-2021 |
| QXP40580 | B.1.617.2 (Delta strain) | 19-08-2021 |
| QXN17606 | B.1.617.2 (Delta strain) | 14-08-2021 |
| QXN15614 | B.1.617.2 (Delta strain) | 13-08-2021 |
| QYY14616 | B.1.617.2 (Delta strain) | 16-08-2021 |

Supplemental table 4: Unique mutation in S and N protein of SARS-CoV-2 in Delta and Delta plus strains

|  | **S protein** | | **N protein** | |
| --- | --- | --- | --- | --- |
| **Sr. No.** | **Mutation** | **Frequency** | **Mutation** | **Frequency** |
| 1. | T19R | 0.9 | A12T | 0.1 |
| 2. | V70F | 0.2 | G18V | 0.1 |
| 3. | T95I | 0.3 | D63G | 0.6 |
| 4. | S112L | 0.1 | R203M | 0.9 |
| 5. | G142D | 0.9 | G215C | 0.3 |
| 6. | E156G | 0.9 | T362I | 0.3 |
| 7. | R214H | 0.1 | D377Y | 0.9 |
| 8. | A222V | 0.3 | R385K | 0.2 |
| 9. | W258L | 0.1 |  |  |
| 10. | K417N | 0.4 |  |  |
| 11. | L452R | 0.9 |  |  |
| 12. | T478K | 0.9 |  |  |
| 13. | D614G | 0.9 |  |  |
| 14. | P681H, P681R | 0.9 |  |  |
| 15. | D950N | 0.8 |  |  |
| 16. | L1063F | 0.1 |  |  |

Supplemental Table 5. Analysis of convalescent COVID-19 T cell reactivity depending on sex.

|  | COVID-19 female | COVID-19 male | P value |
| --- | --- | --- | --- |
| S4 | 4.50±7.79 | 13.00±19.15 | 0.801 |
| S6 | 6.82±6.94 | 8.75±9.75 | 0.580 |
| S15 | 4.68±4.91 | 8.42±10.28 | 0.762 |
| N6 | 5.64±6.19 | 8.75±10.97 | 0.686 |
| N10 | 6.64±7.02 | 9.50±8.04 | 0.481 |
| N19 | 9.59±7.91 | 11.25±10.19 | 0.725 |

Data presented as mean±SD

p value is calculated using Kruskal-Wallis test;

*-p<0.05

Supplemental Table 6. Analysis of aa sequence similarity between S peptides of SARS-CoV-2 and aa sequence at the same location on eCoVs

|  | NL63, S protein; AKT07952 | | 229E, S protein; AWN62679 | | OC43, S protein; APU51916 | | HKU1, S protein; YP_173238 | |
| --- | --- | --- | --- | --- | --- | --- | --- | --- |
|  | location, aa sequence | % similar | location, aa sequence | % similar | location, aa sequence | % similar | location, aa sequence | % similar |
| S1 | 1013-1032 | 65.0 | 830-849 | 60.0 | 1032-1051 | 65.0 | 1036-1055 | 70.0 |
| S2 | 1053-1072 | 55.0 | 870-889 | 55.0 | 1076-1095 | 65.0 | 1072-1091 | 65.0 |
| S3 | 957-964, 968-979 | 40.0 | 774-781, 785-796 | 40.0 | 1274-1293 | 50.0 | 1272-1291 | 40.0 |
| S4 | 941-960 | 35.0 | 758-777 | 35.0 | 978-997 | 40.0 | 974-993 | 45.0 |
| S5 | 929-935, 946-958 | 30.0 | 746-752, 763-775 | 30.0 | 742-761 | 35.0 | 734-742, 762-766, 806-811 | 55.0 |
| S6 | 1036-1055 | 45.0 | 853-872 | 50.0 | 1059-1078 | 70.0 | 1055-1074 | 75.0 |
| S7 | 782-783, 799-816 | 50.0 | 599-600, 616-633 | 45.0 | 829-848 | 40.0 | 826-845 | 40.0 |
| S8 | 1044-1063 | 50.0 | 861-880 | 50.0 | 1067-1086 | 75.0 | 1063-1082 | 75.0 |
| S9 | 988-991, 993-1008 | 35.0 | 805-808, 810-825 | 35.0 | 312-331 | 30.0 |  | 40.0 |
| S10 | 352-354, 361-366, 393-403 | 50.0 | 169-171, 178-183, 210-220 | 40.0 | 1246-1251, 1254-1261, 1285-1290 | 60.0 | 1242-1257, 1259-1262 | 45.0 |
| S11 | 907-917, 930-931, 967-973 | 55.0 | 724-734, 747-748, 784-790 | 55.0 | 991-1110 | 60.0 | 987-1006 | 60.0 |
| S12 | 1090-1109 | 55.0 | 907-926 | 60.0 | 1113-1132 | 50.0 | 1109-1128 | 55.0 |
| S13 | 948-967 | 30.0 | 765-784 | 30.0 | 985-1004 | 50.0 | 981-1000 | 55.0 |
| S14 | 1022-1041 | 55.0 | 839-858 | 55.0 | 1045-1064 | 65.0 | 1041-1060 | 70.0 |
| S15 | 1075-1094 | 40.0 | 892-911 | 40.0 | 859-865, 937-940, 958-961, 968-972 | 70.0 | 1094-1113 | 35.0 |
| S16 | 1052-1070 | 26.3 | 869-887 | 26.3 | 952-955, 961-971, 980-983 | 47.4 | 948-951, 957-971 | 42.1 |
| S17 | 1085-1104 | 50.0 | 902-921 | 60.0 | 1108-1127 | 45.0 | 1104-1123 | 45.0 |
| S18 | 1060-1079 | 60.0 | 877-896 | 55.0 | 1083-1102 | 65.0 | 1079-1098 | 65.0 |
| S19 | 955-956, 963-969, 972-977, 983, 986-989 | 65.0 | 772-773, 780-786, 789-794, 800, 803-806 | 60.0 | 1091-1110 | 35.0 | 1087-1106 | 30.0 |
| S20 | 1187-1206 | 30.0 | 1004-1023 | 30.0 | 628-635, 638-649 | 35.0 | 745-752, 774-785 | 40.0 |
| S21 | 1064-1079 | 31.2 | 881.896 | 31.2 | 1025-1030, 1034-1043 | 37.5 | 1021-1026, 1030-1039 | 37.5 |
| S22 | 1121-1140 | 50.0 | 938-957 | 50.0 | 1144-1163 | 40.0 | 1140-1159 | 40.0 |

.

Supplemental Table 7. Analysis of aa sequence similarity between N peptides of SARS-CoV-2 and aa sequence at the same location on eCoVs.

|  | NL63, N protein; YP_003771 | | 229E, N protein; NP_073556 | | OC43, N protein; YP_009555245 | | HKU1, N protein; YP_173242 | |
| --- | --- | --- | --- | --- | --- | --- | --- | --- |
|  | location, aa sequence | % similar | location, aa sequence | % similar | location, aa sequence | % similar | location, aa sequence | % similar |
| N1 | 327-342, 348-351 | 40.0 | 310-314, 335-339, 359-368 | 30.0 | 321-328, 338-339, 359-368 | 45.0 | 134-143, 153-158, 178-181 | 50.0 |
| N2 | 279-298 | 25.0 | 209-212, 216, 219-224, 230-235, 238-240 | 50.0 | 57-72, 75-78 | 45.0 | 56-75 | 35.0 |
| N3 | 263-274, 277-284 | 35.0 | 132-142, 147-151, 157-160 | 35.0 | 175-194 | 50.0 | 174-190, 198-200 | 60.0 |
| N4 | 263-282 | 40.0 | 278-297 | 30.0 | 299-318 | 35.0 | 297-316 | 35.0 |
| N5 | 9-17, 33, 83-92 | 50.0 | 104-113, 140, 142-144, 201-206 | 50.0 | 245-251, 319-320, 341-351 | 40.0 | 58-62, 69-71, 91-92, 94-97, 111-116 | 40.0 |
| N6 | 272-291 | 40.0 | 287-306 | 40.0 | 308-327 | 45.0 | 306-325 | 40.0 |
| N7 | 244-253, 281-290 | 45.0 | 259-278 | 45.0 | 280-296 | 64.7 | 278-297 | 55.0 |
| N8 | 12-18, 83-92, 102-104 | 60.0 | 3-12, 48-57 | 40.0 | 242-261 | 30.0 | 87-97, 105-113 | 35.0 |
| N9 | 303-307, 310-315, 319-325 | 50.0 | 290-297, 300-305, 309-312 | 50.0 | 420-437 | 27.8 | 113-120, 148-157 | 38.9 |
| N10 | 70-81, 83,85, 122-126 | 50.0 | 72-87, 110-113 | 40.0 | 430-448 | 31.6 | 19-28, 35-40, 44-47 | 45.0 |
| N11 | 263-274, 277-283 | 36.8 | 132-142, 147-151, 157-159 | 36.8 | 175-193 | 52.6 | 174-190, 206-207 | 63.2 |
| N12 | 187-196, 201-205, 210-212 | 44.4 | 9-17, 47, 61-68 | 38.9 | 233-238, 241, 251-252, 271-273, 278-283 | 66.7 | 232-243, 248-253 | 44.4 |
| N13 | 133-140, 144-155 | 50.0 | 206-225 | 40.0 | 183-191, 199-209 | 60.0 | 182-190, 198-208 | 55.0 |
| N14 | 320-337 | 33.3 | 293-310 | 33.3 | 314-331 | 50.0 | 312-329 | 38.9 |
| N15 | 202-209, 215-226 | 35.0 | 163-164, 166-168, 198-199, 209-213, 219-226 | 55.0 | 51-55, 83-84, 91-94, 105-113 | 45.0 | 51-53, --, 54-68 | 44.4 |
| N16 | 143-157, 160-164 | 65.0 | 144-157, 160, 167-171 | 60.0 | 197-209, 213-219 | 60.0 | 196-206, 212-219 | 63.2 |
| N17 | 102-106, 114-116, 125-130, 156-158 | 64.7 | 185-191, 210-216 | 57.1 | 94-97, 127-130, 132, 135-142 | 58.8 | 148-154, 156-160, 171-175 | 47.1 |
| N18 | 186-205 | 35.0 | 185-204 | 30.0 | 229-233, 241-248, 267-273 | 45.0 | 228-232, 240-247, 265-271 | 40.0 |
| N.19 | 174-180, 187-194, 208-213 | 45.0 | 168-170, 185-193, 197-199, 209-213 | 55.0 | 164-183 | 45.0 | 163-182 | 45.0 |

Supplemental Table 8. Analysis of aa sequence similarity between S peptides of eCoVs and aa sequence at the same location on SARS-CoV-2.

|  | SARS-CoV-2, S protein; YP_009724390 | |
| --- | --- | --- |
|  | position of aa sequence | % |
| HKU-1.N1 | 248-256, 328, 355-359, 370-374 | 65.0 |
| HKU-1.N2 | 796-809, 844-849 | 40.0 |
| HKU-1.N3 | 1022-1026, 1050-1062 | 50.0 |
| 229E.N1 | 782-789, 811-821 | 40.0 |
| 229E.N2 | 805-823 | 52.6 |
| 229E.N3 | 1047-1057, 1091-1099 | 40.0 |
| OC-43.N1 | 349-352, 355-356, 384, 389-398 | 52.9 |
| OC-43.N2 | 653-656, 663, 697-699, 701-711, 738-739 | 50.0 |
| OC-43.N3 | 291-295, 301-304, 320-321, 332-338, 349-350 | 55.0 |
| NL63.N1 | 824, 828-830, 843-848, 853-856, 862-863, 884, 898-901 | 70.0 |
| NL63.N2 | 973-992 | 40.0 |
| NL63.N3 | 312-330 | 36.8 |

% - percent of identity

Supplemental Table 9. Analysis of aa sequence similarity between N peptides of eCoVs and aa sequence at the same location on SARS-CoV-2.

|  | SARS-CoV-2, N protein; YP_009724397 | |
| --- | --- | --- |
|  | position of aa sequence | % |
| HKU-1.N1 | 234-236, 245-261 | 35.0 |
| HKU-1.N2 | 69-73, 80-94 | 45.0 |
| HKU-1.N3 | 312-316, 325-334, 339-344 | 45.0 |
| 229E.N1 | 247-256, 258-262, 283-287 | 50.0 |
| 229E.N2 | 251-268 | 38.9 |
| 229E.N3 | 158-163, 180-191, 194-195 | 60.0 |
| OC-43.N1 | 66-76, 120-122, 134-139 | 60.0 |
| OC-43.N2 | 102-105, 128-143 | 40.0 |
| OC-43.N3 | 95-105, 120-128 | 40.0 |
| NL63.N1 | 92-97, 104-106, 113-116, 126-131 | 52.6 |
| NL63.N2 | 136-139, 148-150, 179-182, 192-200 | 50.0 |
| NL63.N3 | 280-289, 293-294, 299, 329, 333-334, 360-362 | 47.4 |

% - percent of identity

Supplemental Table 10. Analysis of COVID-19 convalescent serum reactivity with eCov S and N peptides.

|  | COVID-19 | Control 2020 | P value |
| --- | --- | --- | --- |
| NL63-N1 | 0.23±0.14 | 0.21±0.09 | 0.865 |
| NL63-N2 | 0.20±0.11 | 0.17±0.06 | 0.242 |
| NL63-N3 | 0.21±0.10 | 0.19±0.07 | 0.384 |
| NL63-S1 | 0.21±0.11 | 0.18±0.06 | 0.189 |
| NL63-S2 | 0.20±0.10 | 0.17±0.06 | 0..122 |
| NL63-S3 | 0.21±0.10 | 0.19±0.07 | 0..599 |
| OC43-N1 | 0.20±0.09 | 0.18±0.06 | 0..227 |
| OС43-N2 | 0.21±0.11 | 0.21±0.12 | 1..000 |
| OC43-N3 | 0.24±0.14 | 0.22±0.09 | 0..819 |
| OC43-S1 | 0.21±0.14 | 0.20±0.08 | 0..677 |
| OC43-S2 | 0.21±0.10 | 0.20±0.09 | 0..511 |
| OC43-S3 | 0.33±0.27 | 0.35±0.20 | 0..182 |
| HKU1-N1 | 0.33±0.21 | 0.34±0.24 | 0.616 |
| HKU1-N2 | 0.30±0.23 | 0.28±0.19 | 0.629 |
| HKU1-N3 | 0.22±0.24 | 0.17±0.19 | 0.053 |
| HKU1-S1 | 0.66±0.36 | 0.92±0.48 | **0.010*** |
| HKU1-S2 | 0.25±0.22 | 0.23±0.22 | 0.344 |
| HKU1-S3 | 0.18±0.20 | 0.16±0.20 | 0.269 |
| 229E-N1 | 0.26±0.22 | 0.26±0.27 | 0.511 |
| 229E-N2 | 0.28±0.22 | 0.27±0.19 | 0.739 |
| 229E-N3 | 0.27±0.21 | 0.22±0.18 | 0.165 |
| 229E-S1 | 0.25±0.23 | 0.21±0.19 | 0.344 |
| 229E-S2 | 0.30±0.26 | 0.25±0.23 | 0.286 |
| 229E-S3 | 0.25±0.21 | 0.22±0.19 | 0.546 |

Data presented as mean±SD

p value is calculated using Kruskal-Wallis test with BH adjustment; *-p<0.05
